# Phase Imaging Methods in the Scanning Transmission Electron Microscope

**DOI:** 10.1021/acs.nanolett.4c06697

**Published:** 2025-06-28

**Authors:** Gabriel Sanchez-Santolino, Laura Clark, Satoko Toyama, Takehito Seki, Naoya Shibata

**Affiliations:** † GFMC, Departamento de Física de Materiales & Instituto Pluridisciplinar, 213131 Universidad Complutense de Madrid (UCM), 28040 Madrid, Spain; ‡ School of Physics Engineering and Technology, University of York, York YO10 5DD, U.K.; § Institute of Engineering Innovation, School of Engineering, The University of Tokyo, Yayoi 2-11-16, Bunkyo-ku, Tokyo 113-8656, Japan; ∥ PRESTO, Japan Science and Technology Agency, Kawaguchi, Saitama 332-0012, Japan; ⊥ Nanostructures Research Laboratory, Japan Fine Ceramics Center, 2-4-1, Mutsuno, Atsuta, Aichi 456-8587, Japan; # Quantum-Phase Electronics Center (QPEC), The University of Tokyo, Hongo 7-3-1, Bunkyo-ku, Tokyo 113-8656, Japan

**Keywords:** scanning transmission electron microscopy, phase imaging, differential phase contrast, ptychography, 4D-STEM

## Abstract

Scanning transmission electron microscopy (STEM) has become an essential tool for investigating materials, providing detailed characterization at nano and atomic scales. By combining subangstrom resolution Z-contrast imaging with analytical X-ray and electron spectroscopies, STEM allows the visualization of atomic arrangements, crystal defects, and interfaces, understanding sample properties and correlating these with the functionalities of materials and devices. Recent advancements in phase imaging techniques, including differential phase contrast (DPC) and electron ptychography, have further enhanced STEM capabilities. These methods allow direct imaging of electromagnetic fields, the study of beam-sensitive materials with high dose efficiency, or resolving the 3D structure of materials, proving invaluable for investigating intricate nanoscale phenomena. This review introduces phase imaging methods in the STEM and explores how the most recent innovations are driving progress in nanoscience, deepening material insights and shaping next-generation applications in electronics, energy storage, and catalysis.

## Graphical abstract

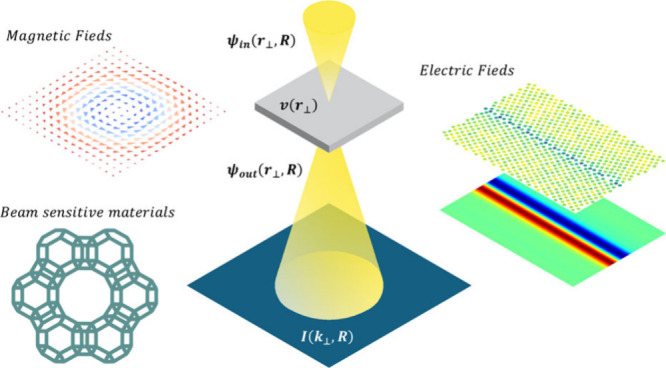

Scanning transmission electron microscopy (STEM) has emerged as one of the most powerful techniques in the field of nanoscience, offering an unparalleled view into the structure and properties of materials at the nano and atomic scales. In STEM, a finely focused probe is rastered over the sample, enabling the simultaneous acquisition of multiple analytical signals with single atom sensitivity.^1,2^ It is this versatility which makes STEM an essential technique for the characterization of nanomaterials by providing detailed insights into their physical, chemical, and electronic characteristics. One of the most powerful aspects of STEM is the ability to obtain easily interpretable incoherent annular dark field (ADF) images^3,4^ depicting a contrast related to the atomic number (Z-contrast). The combination of Z-contrast with the subangstrom resolution of modern aberration corrected instruments^5,6^ have provided the community with the capabilities needed to visualize atomic arrangements, crystal defects, and interfaces within nanoparticles, nanowires, thin films, or two-dimensional (2D) materials.

On the other hand, it is in the past decade that coherent phase contrast imaging methods for STEM have begun to show their potential. Annular bright field (ABF) imaging, first suggested by H. Rose, uses phase contrast in the STEM to improve the visualization of light atomic columns.^7,8^ More recently, advances in detector technologies and computing power have driven significant progress in the development of new and innovative phase imaging techniques, such as atomic-resolution differential phase contrast and electron ptychography,^9–11^ paving the way for a new revolution in the field. The use of aberration-corrected probes has also enhanced these coherent imaging techniques due to both the increased probe currents and the possibility to open the probe-forming aperture (convergence angle) to larger angles while maintaining coherent imaging conditions, leading to higher detectable contrast. Phase contrast techniques provide capabilities that are not available in conventional imaging modes, as they not only allow us to visualize materials, but also to acquire information about the object’s electrostatic potential, the presence of electric and magnetic fields, and can be used to perform highly dose-efficient experiments for the characterization of beam sensitive materials.

For the study of nanomaterials and nanodevices, in which functional properties are determined by local modulations of the atomic arrangement or the presence of confined electromagnetic fields at small active regions, such as grain boundaries, interfaces or point defects, these developments have been particularly impactful. For instance, in semiconductor nano devices such as field-effect and high-mobility transistors, where their electronic characteristics are determined by the physical properties of heterointerfaces, differential phase contrast has enabled the direct visualization and study of two-dimensional electron gas formation.^12^ In an alternative application, highly dose-efficient phase imaging methods have enabled imaging of the complete atomic structure of materials, including light (low Z) elements, with minimal beam-induced damage, which is key for the study of beam-sensitive materials such as Li-rich battery cathode materials.^13^ These highlight how phase imaging techniques in STEM can offer essential insights into critical features of materials, understanding their role at the atomic and nanoscale and thereby optimizing their impact on performance and functionality of new materials and technologies across various fields, including electronics, energy storage, medicine, and catalysis. The advancements in nanoscience and scanning transmission electron microscopy are occurring simultaneously, with each driving the other forward. In this work, we provide an overview of how recent progress in phase imaging techniques, particularly differential phase contrast and ptychography, have shaped current research on nanomaterials and devices and delve into the possibilities these techniques will bring in the future.

## Basic Principles of Differential Phase Contrast

Differential phase contrast (DPC) imaging was initially proposed by Dekkers and de Lang^14^ as a method to enhance phase contrast in STEM. It was later demonstrated that this signal represents the gradient of the object potential, and thus could be used to detect electric and magnetic fields within materials.^15,16^ The principle of DPC stems from the interaction of electrons, as charged particles, with the Coulomb and Lorentz forces generated by electromagnetic fields within materials. As waves, the phase of the incident electron wave function undergoes a shift proportional to the field’s strength. Equivalently, as particles, electrons are deflected at an angle proportional to the field’s intensity. These effects can be measured by using segmented or pixelated detectors, which are sensitive to the spatial distribution of scattered electrons across the detector plane, as illustrated in Figure 1. In this way, one can measure the deflection of the electron beam at each position of the raster scan across the sample, and from this deflection, infer the presence of local electromagnetic fields.

**Figure 1:**
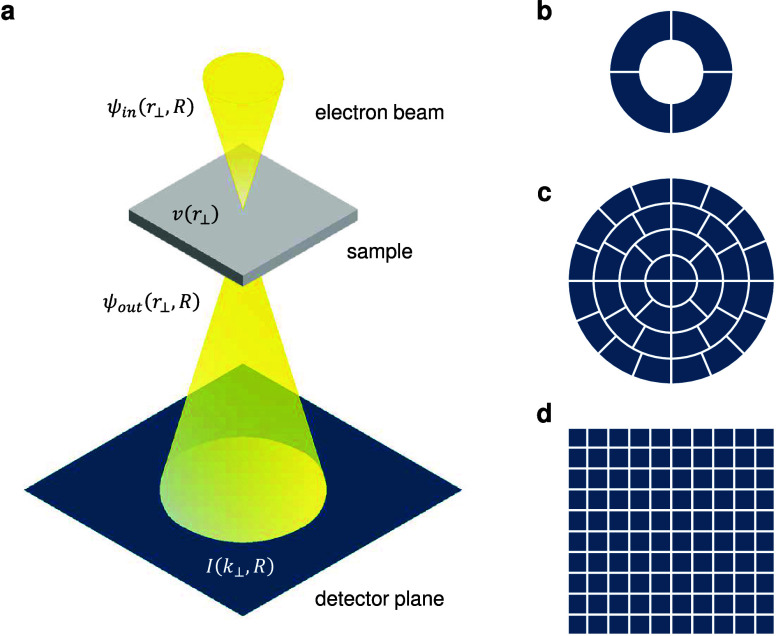
Schematic illustration of phase contrast imaging methods and detectors in the STEM. (a) Schematic of the STEM optical system including the incident electron beam, the sample which produces a deflection (shift) of the beam (phase), and the detector plane where the data is collected. Here *r*_⊥_ denotes coordinates perpendicular to the optical axis, *k*_⊥_ denotes the Fourier space coordinate conjugate to *r*_⊥_, and *R* denotes the position of the STEM probe on the specimen surface. (b) 4-fold segmented detector. (c) 40-fold segmented detector. (d) Pixelated 4D-STEM detector.

Using a segmented detector, this is achieved by measuring the differential signal between two diametrically opposed segments of the detector and converting the signal into field vectors. When the probe size is smaller than the scale over which the electric or magnetic fields vary, the DPC image contrast arises from the beam deflection and is essentially proportional to the strength of the field.^17^ The signal is optimally detected with in-focus conditions, and hence, DPC imaging is compatible with simultaneous Z-contrast imaging, unlike Lorentz TEM (where large defocus is usually needed to obtain phase contrast) or holography (which requires nonstandard optical settings). This characteristic makes DPC-STEM particularly suitable for correlative analysis of structural and magnetic or electric field features such as the built-in electric field at a p–n junction^18^ or magnetic spin textures.^19^

In 2012, Shibata et al.^9^ showed the possibility to perform atomic-resolution DPC experiments and detect the atomic electric fields. This and subsequent works^17,20^ demonstrated that the conventional understanding of a rigid deflection of the beam disk due to electric and magnetic fields was overly simplistic as the complex interaction of the electron probe with the sample produces an inhomogeneous distribution of intensity at the diffraction plane. This effect is enhanced at atomic resolutions or when the probe is of similar size to the scale of the gradient of the electrostatic potential. To overcome this challenge, one can use a detector with more segments or pixels, as shown in Figure 1 (c) and (d), which allows a more accurate determination of the center of mass (COM) of the transmitted beam’s intensity distribution at the diffraction plane. This signal more accurately reflects the interaction between the incident electron beam and the specimen’s potential and, within the phase object approximation, exhibits a linear relationship with the gradient of the phase, i.e. the electric or magnetic field. This idea was first proposed by Waddell and Chapman^16,21^ and later demonstrated experimentally thanks to technological advances in multi segmented and pixelated 4D-STEM detectors.^22,23^

Nevertheless, the interpretation and quantification of differential phase contrast signals to detect electromagnetic fields in materials requires the consideration and understanding of complex phenomena, such as multiple electron scattering or dynamical diffraction, necessitating careful analysis.^24^ DPC images can be affected by diffraction contrast in crystalline samples, similar to what is observed in conventional bright-field (BF) STEM images while other factors such as sample thickness, tilt, and defocus effects can all impact the clarity and accuracy of the measurements. This has been extensively addressed in recent years: different interpretations of the DPC signal based on quantum mechanical^22^ and linear imaging theory^25^ have been proposed, and the effect of different phenomena on DPC signals such as partial spatial coherence, plasmon scattering, sample tilt etc., have been also discussed.^26,27^ Some of these can be overcome experimentally, for example, diffraction contrast can be minimized by tilting the specimen off-axis or by acquiring tilt DPC image series.^28^ Moreover, several studies have used comparisons with density functional theory calculations to aid the interpretation of DPC signals, allowing the understanding of how S vacancies alter the local chemical state of transition metal dichalcogenide two-dimensional materials^29^ or revealing charge variations at anionic electron sites in electrides.^30^

## Basic Principles of Ptychography

With the increasing availability of high-speed segmented and pixelated detectors, with acquisition times comparable to conventional STEM detectors,^31,32^ new methods of imaging via computational algorithms become feasible in STEM. These are collectively known as four-dimensional STEM (4D-STEM). Within 4D-STEM methods,^33^ perhaps the group of methods to have found the widest application is ptychography (“tie-cog-raphy”). By applying ptychographic algorithms, and with some knowledge of the optical setup (typically: probe size, probe step-size, convergence angle, camera length and detector pixel size and arrangement), one can deconvolve sample and microscope effects from within the collected data set to reconstruct the transmission function(s) of the sample (proportional to the projected electromagnetic potential). Examples of ptychographic imaging, compared to conventional STEM imaging approaches are illustrated in Figure 2.

**Figure 2:**
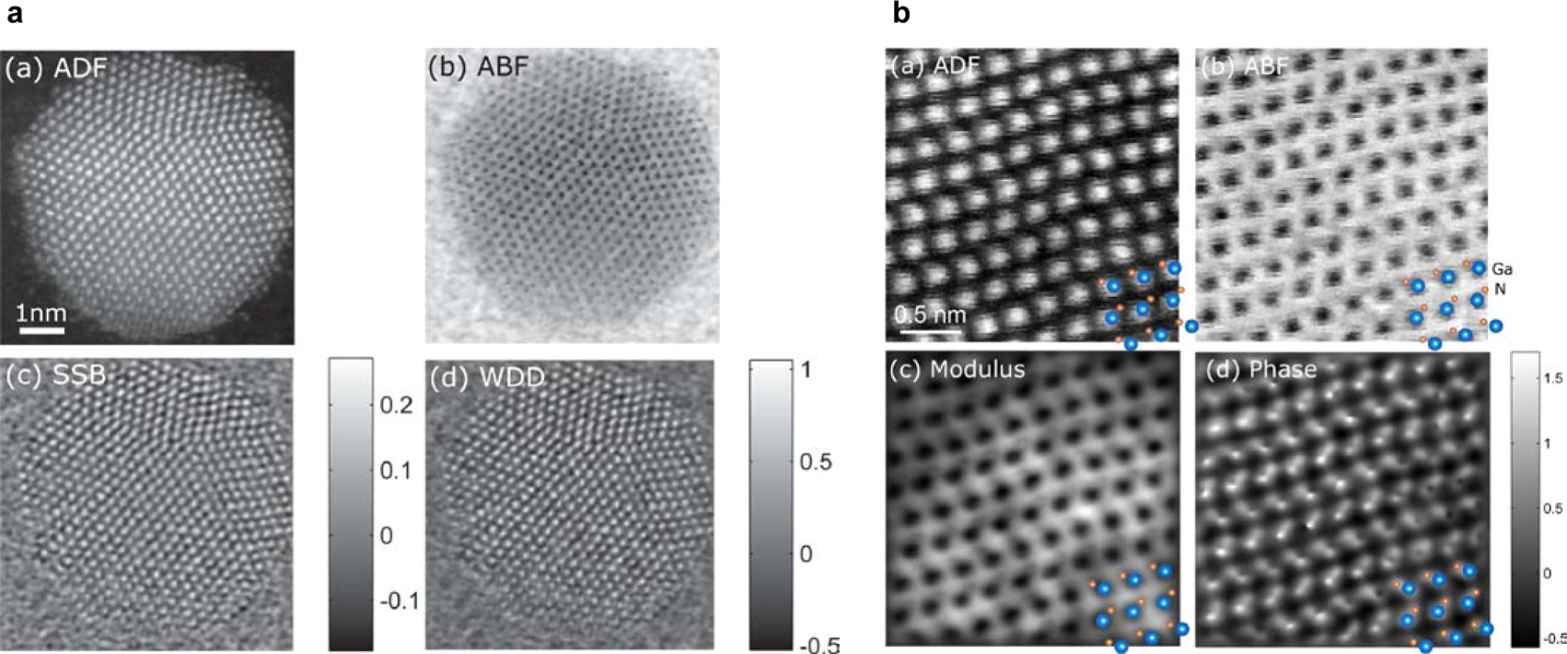
Conventional STEM compared to ptychographic methods. (a) Imaging a gold nanoparticle on a thin carbon support using simultaneous ADF, ABF, and ptychography SSB phase and WDD phase. Color bar in radians. (b) Simultaneous ADF, synthetic ABF, and the reconstructed modulus and phase of bulk GaN imaged along [2110]. Panels (a) and (b) adapted from [^37^]. Available under a CC-BY 4.0 license. Copyright 2017 Elsevier.

On the left-hand side of Figure 2, a gold nanoparticle is imaged using two conventional STEM approaches, and two ptychographic imaging approaches. In this arrangement, the interpretation of the ptychographic phase image follows as per conventional STEM image interpretation, while noting that there are specific advantages to the ptychographic approach, discussed later in this section.

On the right-hand side of Figure 2, a region of GaN is imaged via conventional STEM, alongside the modulus and phase from a ptychographic reconstruction. We note that in this case, the phase map enables the lighter element to be imaged more clearly than with conventional approaches - a key advantage of ptychography. This image also highlights some challenges of interpreting ptychographic imaging: the sharp black-white boundaries seen on some columns in the phase map are a phase wrapping artifact (discussed further in Clark et al.^34^).

In certain cases, this can be advanced further to handle data sets with imprecise *a priori* knowledge, to give super-resolution images or to reveal depth-sensitive sample information - these approaches will be discussed in more detail below. These data become available in addition to the structural and functional information which can be extracted from the same data set, and in a manner that makes efficient use of the input electron fluence - therefore finding further application in the characterization of beam-sensitive materials (discussed further below).

Ptychography is increasingly widely used across imaging regimes from X-ray to ultraviolet, and with photons, neutrons and electrons.^35,36^ As such there are a vast array of methods and variations in community nomenclature in literature. Herein, we restrict our discussion to an overview of methods and applications well-suited to use in STEM-ptychography. To build an ordered understanding of the currently available STEM-ptychography methods, we make an initial comparison between iterative and direct (i.e., noniterative) methods.

The direct methods require one to begin with a 4D-STEM data set collected in a conventional high-resolution STEM alignment, in which the STEM detector is replaced with a pixelated detector, shown in Figure 1 (d). In general, the probe focus is ideally set to the midplane of the specimen^34,36^ and the camera length should be set such that the bright-field disc fills a significant fraction of the detector field of view (approximately 1/3 to 1/2), although this can be dependent on the experiment. The probe step size (real space distance between probe positions in the STEM scan) must be sufficiently small, at least matching Nyquist sampling, to ensure features are sampled. In this setup it is often convenient to note that this is very similar to the ideal collection conditions for, for example, HAADF-STEM or EDX imaging - which can therefore be performed completely simultaneously for perfect data coregistration - as with DPC-STEM discussed above.

With this 4D-STEM data set, one can then choose between the available analysis methods: single sideband (SSB) or Wigner distribution deconvolution (WDD). Both of these approaches make the multiplicative object approximation (that the sample is sufficiently thin that beam propagation is negligible within the sample), but are differentiated in the severity of their subsequent approximation: in SSB, one further makes the weak phase object approximation (assuming that the sample affects only the phase but not the intensity of the transmitted beam – and that the phase change imposed on the beam by the sample is much less than 1 rad). As such - one might be concerned that these methods are only valid for impractically thin and weakly scattering samples - but in practice that has not proven to be the case.^34,37^ Despite their different underlying approximations, in practical examples it has not been demonstrated that WDD is more widely applicable or stable than SSB. Indeed, in SSB there are no free parameters required for the experimenter to choose - all can be defined from optical conditions, while in WDD one has a free choice of parameter in the Wiener filter step when performing the Wigner deconvolution - and careful choice here is needed.^38^

In the iterative methods - there are a broad range of choices the experimenter can make, and few immovable parameters. This can lead to high-resolution output data from challenging samples - but can also leave open questions as to how the resultant image was verified and must be handled carefully. The first practical demonstrations of iterative ptychography follow what is now known as the ptychographic iterative engine (PIE) method by Faulkner and Rodenburg,^39,40^ where a relatively large probe illuminates the sample (large either due to a very small convergence angle, or due to deliberately applied defocus), a far-field diffraction pattern collected, the probe shifted and the process repeated as above, then solved through a serial implementation of Gerchberg-Saxton-type phase retrieval. In these methods, one must ensure that the neighboring probe positions overlap on the sample by a reasonable proportion of around a 70 to 80%^41^ to ensure stable convergence of the global solution. In such methods, the probe size and step size are not the limiting factor of the resolution - which is instead determined by a combination of these, along with the sampling in the detector plane. Accordingly, rather few probe positions may be needed to interrogate a sample field-of-view – which may present a dose-efficient route to a particular materials problem but cannot be obtained simultaneously with conventional STEM data sets.

Further developments followed from this initial iterative framework. In 2009, extended PIE (ePIE) was introduced^42^ enabling one to solve for both the object and the probe simultaneously. Other developments have addressed both algorithm development, such as the conjugate gradient and difference map approaches^43,44^ and remains an active field of research. For further detail, we refer the reader to the literature.^35,45^

## Electric Field Imaging Applications

Phase imaging techniques in STEM have enabled the visualization of electric fields, potentials, and charge density distributions from the nanoscale down to the subatomic level. Since the practical application of DPC-STEM imaging for electric field imaging in GaN-related semiconductor quantum wells^46^ and ferroelectric polarization fields^9^ was reported in the early 2010s, these techniques have been widely applied to various material systems. This section highlights how phase imaging reveals electric fields at both the nanoscale and atomic scale.

Nanoscale electric fields and charges play a critical role in many material applications, prompting extensive investigations via phase imaging. For example, DPC-STEM and ptychography have successfully visualized electric fields in semiconductor p–n junctions^17,18,27,47^ and polarization electric fields in GaN-related semiconductor quantum wells.^48^ In the latter case, Muller et al. proposed an approach to extract polarization fields by unit-cell averaging of atomic-resolution DPC-STEM electric field maps using Voronoi cells, effectively canceling the electric field contributions from atomic nuclei and extracting the polarization field.

In contrast to the Muller approach, the observation of nanoscale electric fields often employs convergent electron probes with semiconvergence angles below 1 mrad, in relatively thick samples (occasionally exceeding 100 nm). This configuration, sometimes referred to as “Lorentz mode”, arises from balancing spatial resolution with electromagnetic field sensitivity in DPC-STEM. Such settings are particularly important for detecting relatively weak, nanoscale electric fields, which differ from strong atomic-scale electric fields originating from positive atomic nuclei and surrounding electron clouds. However, these conditions may introduce strong dynamical effects (diffraction contrast), which can obscure electric fields at crystal interfaces and defects. To address these issues, the tilt-scan averaged DPC (tDPC) STEM technique was recently developed.^28^ In tDPC-STEM, the electron beam is systematically tilted at multiple angles and the resulting diffraction patterns are averaged to effectively mitigate dynamical effects. Using tDPC-STEM, Toyama et al. quantitatively visualized the two-dimensional electron gas (2DEGs) at the GaN/Al_*x*_In_1–*x*_N heterointerfaces- key structures in high-electron-mobility transistors. Their study successfully quantified differences in 2DEG distributions at GaN/Al_*x*_In_1–*x*_N heterointerfaces with varying compositions, achieving excellent agreement with Poisson-Schrödinger simulations.^12^

Furthermore, a recent study has extended the application of tDPC-STEM to observe charge distributions at grain boundaries in Yttria-Stabilized Cubic Zirconia (YSZ), a solid electrolyte used in solid oxide fuel cells.^49^ Figure 3 (a)-(d) show electric field images and line profiles at ∑5(310) and ∑5(210) grain boundaries. Divergent electric fields were detected exclusively at the ∑5(310) grain boundary, suggesting the presence of the positively charged grain boundary core and negative space charges. This finding correlates with the stronger yttrium segregation observed at ∑5(310) grain boundaries compared to ∑5(210) grain boundaries, highlighting how grain boundary plane, core atomic structure, and dopant segregation collectively influence local charge behavior.

**Figure 3:**
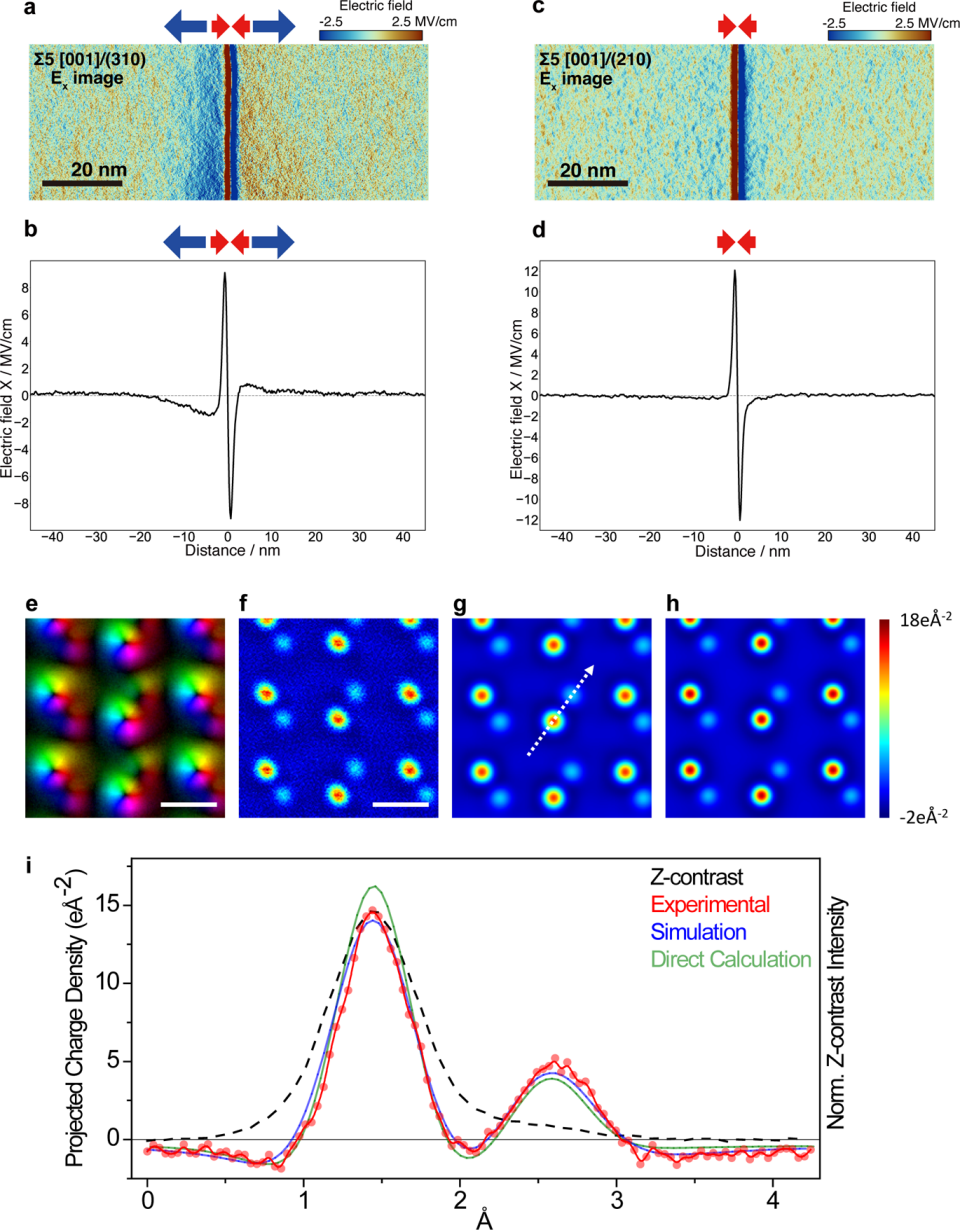
Electric field imaging applications. (a) Horizontal-component electric field (Ex) map and (b) horizontal-component electric field line profile of a ∑5[001]/(310) yttria-stabilized cubic zirconia grain boundary. (c) Horizontal-component electric field map and (d) horizontal-component electric field line profile of a ∑5[001]/(210) yttria-stabilized cubic zirconia grain boundary. In the electric field maps in (a) and (c), blue color indicates the leftward and red color indicates the rightward electric fields. In the line profiles in (b) and (d), the leftward electric field is defined as the negative value and the rightward electric field defined as the positive value. (e) Unit-repeated-averaged image of the projected electric field for GaN down the [1120] direction. The color and brightness, respectively, denote the direction and magnitude of the field. (f) Projected total charge density map calculated from (e). The scale bars correspond to 2 Å. (g) Projected total charge density map obtained from scattering simulations. (h) Projected total charge density calculated directly from the isolated-atom form factors, convolved with the probe intensity. (i) Line profiles taken along the white arrow in (g) showing the normalized Z-contrast signal profile (dashed black line) and the experimental (red dots), simulated (blue), and calculated (light green) projected total charge density profiles. Negative (positive) values represent negative (positive) charge densities. Panels (a)–(d) reproduced with permission from [^49^]. Copyright 2024 Springer Nature. Panels (e)–(i) reproduced from [^53^]. Copyright 2018 American Chemical Society.

On the atomic-scale electric field imaging, employing a larger convergence angle, and hence subangstrom electron probes, enables the visualization of local electric fields arising from positive atomic nuclei and electron clouds. Such atomic-scale electric field mapping was first demonstrated in perovskite oxides such as SrTiO_3_ and BaTiO_3_.^9,22^ Further advancements include the observation of electric fields in single atoms,^23^ two-dimensional materials at low-acceleration voltage,^50–52^ and the visualization of bonding charge distributions between atomic nuclei using DPC-STEM and ptychography.^29,30,53–55^ Figure 3 (e)-(i) show the results of atomic-resolution DPC-STEM imaging of a GaN crystal along the [1120].^53^ The electric field map allows experimental determination of the total charge density using Gauss’s law, as shown in Figure 3 (i), (f). By comparing this experimental result with both dynamical scattering simulations and direct charge density calculations from isolated atomic form factors (Figure 3 (g)-(h)), the authors confirmed the possibility of detecting the negative charges (shown in dark blue) surrounding the positive nuclear charges at Ga sites (shown in deep red). The consistency of these results across experiments, simulations, and direct calculations imply the sensitivity of the technique to the electron clouds, resolving the negative and positive charge density distributions in real space. Additionally, line profiles of the charge density map provided a quantitative comparison among the three methods, demonstrating that the atomic-resolution DPC-STEM can directly and quantitatively visualize electron clouds at the atomic scale.

## Magnetic Field Imaging Applications

DPC-STEM has become an indispensable tool for studying magnetic structures at the nanoscale as well as electric field mapping. After the theoretical proposal of DPC STEM,^7,14,15^ Chapman and colleagues conducted pioneering experiments in the 1970s to observe magnetic domains.^16^ This provided crucial experimental validation of the theoretical concepts and showcased the potential of DPC-STEM in magnetic imaging. While significant advancements were made,^56,57^ the technique was not widely adopted by the broader scientific community during that period, possibly because STEM had not been commonly used for imaging for some time. However, with the rapid advancement of aberration correction technology since the early 2000s, STEM became widely adopted in materials analysis, and the DPC method began to attract renewed attention.

The integration of magnetic field observation using DPC-STEM with local atomic structure analysis using aberration-corrected ADF-STEM has become possible, enabling comprehensive studies of materials at the atomic level. This synergy allows for the investigation of atomic structures at interfaces and their magnetic coupling. For example, in Fe_3_O_4_, experimental identification of local structures and magnetic coupling at three types of twin boundaries has been carried out.^58^ Revealing the nature of magnetic coupling across grain boundaries and interfaces can lead to a better understanding of the magnetic behaviors of polycrystalline magnetic materials, greatly benefiting the development of advanced magnetic materials and devices.

Phase imaging methods in the STEM have also been applied to the direct observation of magnetic skyrmions—unique vortex-like magnetic structures discovered in recent years that are potential candidates for future memory devices. DPC-STEM and ptychography have been used for visualizing skyrmions lattice structures such as domain boundary cores^59^ and edge dislocations.^60^ Moreover, it has been observed that skyrmions can be controlled by creating surface defects.^19^ Figure 4a shows skyrmions confined within artificially fabricated triangular enclosures created by electron beams. Observing skyrmions in localized regions provides important insights into challenges that may arise from interactions with lattice defects and into methods for controlling skyrmions through surface processing. This knowledge is crucial for the development of skyrmion-based devices and for overcoming potential obstacles in their practical applications.

**Figure 4:**
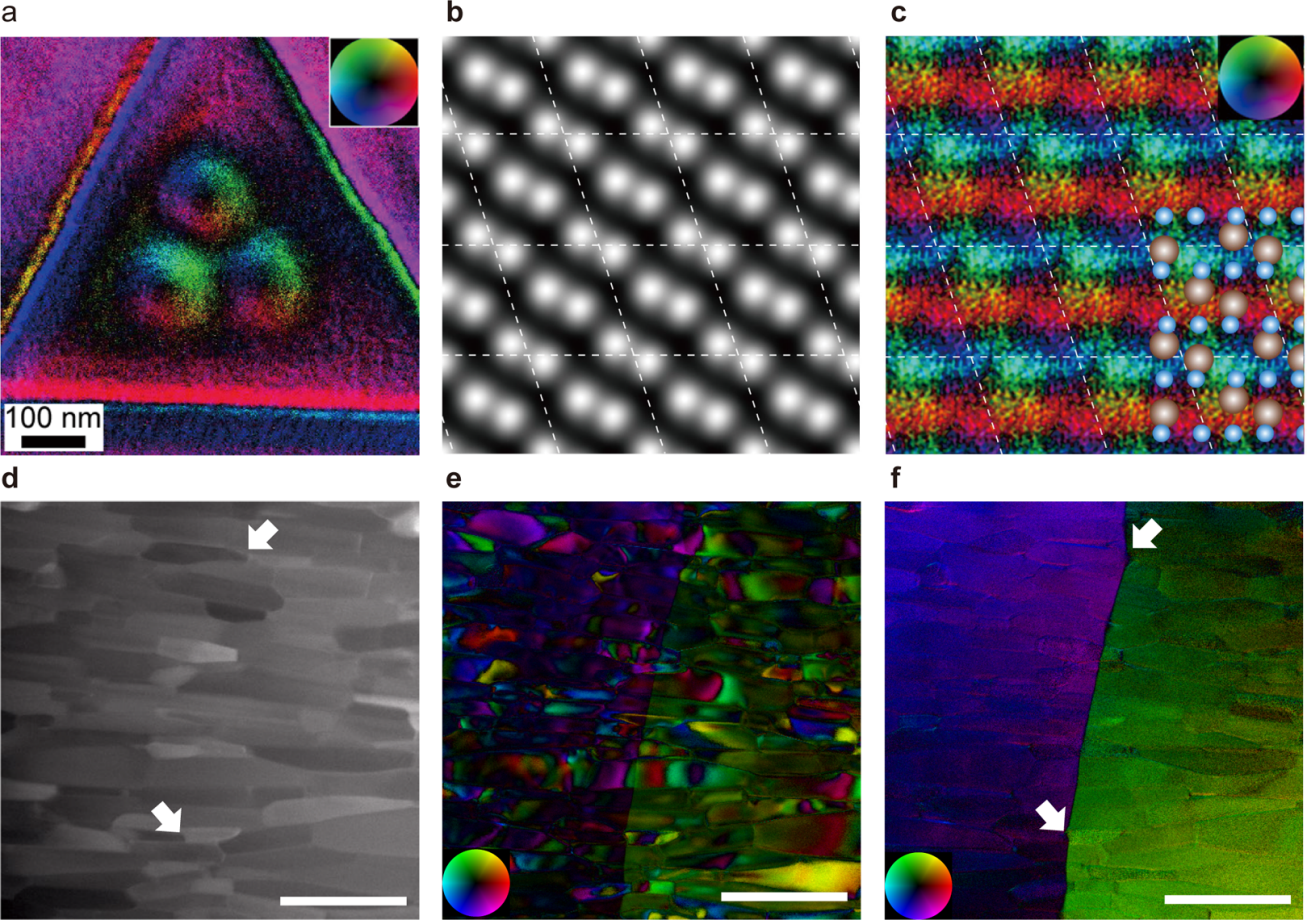
Magnetic field imaging applications. (a) In-plane magnetic field vector map. A stable triple-skyrmion state was observed under the residual field of the objective lens (∼20 mT). (b) ADF image and (c) magnetic field map of hematite (α-Fe_2_O_3_) constructed from the unit-cell-average and tiled magnetic DPC images. (d) HAADF, (e) conventional DPC, and (f) tDPC images of Nd–Fe–B polycrystalline magnet. Panel a reproduced from [^19^]. Copyright 2018 American Chemical Society. Panels (b) and (c) reproduced with permission from [^61^]. Copyright 2022 Springer Nature. Panels (d)–(f) reproduced with permission from [^62^]. Copyright 2024 Springer Nature.

Advancements in DPC-STEM have also led to magnetic field observations at the atomic scale using atomic-resolution probes.^61^ The antiferromagnetic order in Fe_2_O_3_ has been visualized (Figure 4 b, c). In atomic-resolution DPC imaging, atomic electric fields are primarily observed, but when antiferromagnetic order is present, magnetic field signals overlap with the atomic electric field signals, which is extremely weak—less than 1% of the signal from the atomic electric field. In Figure 4c, the antiferromagnetic order is visualized by applying a kernel filter that cancels out the atomic electric field signals from multiple adjacent atomic columns, thereby extracting only the magnetic field signals. This result demonstrates the potential to quantify the magnetization of each atomic column, even at defects such as interfaces. Since the atomic magnetic field imaging method using a kernel filter requires prior knowledge of the magnetic ordering, further methodological developments are necessary for analyzing unknown magnetic structures. However, when several predicted magnetic structures are available, applying kernel filters specifically designed for these structures may enable effective magnetic structure analysis. Observing magnetic fields at the atomic scale may lead to a better understanding of the behavior of spintronic devices, as it provides insights into magnetic configurations at the most fundamental level.

Despite these advances, significant challenges remain in magnetic field observation using DPC-STEM. As with electric field observations, diffraction contrast can negatively impact DPC images, creating artifacts that limit observations to single crystals or samples with minimal strain. However, many permanent magnets have polycrystalline structures, and spintronic devices often feature thin-film architectures. Therefore, magnetic field observation of these samples is highly important. To overcome these limitations, the tDPC method has been adopted and the scope of magnetic field observations has been expanded, similarly to its application in electric field studies. Figure 4 d-f show the results of observing Nd–Fe–B magnets^62^ using both conventional DPC and tDPC methods. The conventional DPC method fails to clearly reveal magnetic domain structures due to interference from diffraction contrast (Figure 4e). In contrast, the tDPC method successfully uncovers distinct magnetic domain structures (Figure 4f). By utilizing this method, researchers expect to elucidate the mechanisms underlying coercivity in permanent magnets. In summary, DPC-STEM has evolved significantly, enabling magnetic field observations from the microscale down to the atomic scale. The integration with aberration-corrected STEM techniques, such as ADF, EDS, and EELS, allows for simultaneous analysis of atomic structures and magnetic properties, providing comprehensive insights into material behaviors.

## Study of Beam Sensitive Materials

STEM is a key tool in materials science, providing high-resolution imaging and enabling the direct observation of atomic structures, thanks to the advances in aberration-corrected probes, allowing for the use of larger convergence angles. Conventional imaging modes such as HAADF and ABF imaging, which rely on single annular detectors, are widely adopted for atomic-resolution observation. However, for materials that are highly sensitive to electron beams – such as zeolites, battery materials, metal–organic frameworks (MOFs), two-dimensional (2D) materials, organic materials, and biological specimens – these techniques often prove insufficient. Energetic beams of electrons can cause structural damage,^63^ which has made high-resolution TEM a more suitable alternative for some of these systems in the past due to its higher dose efficiency, or the ability to achieve a higher signal-to-noise ratio (SNR) with the same electron dose.

The development of novel phase imaging techniques for the STEM and the use of new detectors able to collect scattered electrons with position sensitivity in reciprocal/momentum space (whether radially or annularly segmented, or on a pixelated array detector) – now allows a more careful and sensitive analysis of the detected information. HAADF and other ADF techniques utilize the dark-field region, which can be dose-efficient for heavy elements.^64^ However, as these methods provide little signal for light elements, an alternative approach is required to achieve dose-efficient imaging across all elements, including light ones. For a more comprehensive dose-efficient imaging strategy, we instead seek coherent imaging methods that utilize finely divided detectors. These information-rich data sets can be analyzed through a range of computational imaging algorithms, each of which take a different approach to maximizing the sample information revealed in the final image. Key tools in this category include ptychographic techniques such as SSB ptychography^65^ or optimal bright field (OBF) imaging,^66^ and integrated differential phase contrast (iDPC) or integrated center of mass (iCOM) imaging.^67^ The utility of these methods for beam-sensitive materials is based around their dose-efficient image contrast mechanisms: conventional STEM images are formed by simply collecting the total number of electrons scattered to a particular angular range at each probe position. The use of multiple detectors enables the details of the scattering to be interrogated in a much more fine-grained approach.

In this context, the concept of improving dose efficiency in STEM was first highlighted through SSB ptychography.^10^ This technique demonstrated high dose efficiency, with the successful visualization of graphene. Ptychography has also been emloyed to investigate beam sensitive Li-ion battery materials, Figure 5 a, b shows atomic-resolution images of a Li_1.2_Mn_0.6_Ni_0.2_O_2_ particle: ADF image (Figure 5a) and WDD ptychographic reconstruction (Figure 5b). This result indicates the ptychographic reconstruction exhibits a much higher SNR than the ADF image. Another dose-efficient approach involves using iDPC or iCOM techniques. By integrating the electric field information obtained through DPC, one can reconstruct electrostatic potential maps with high SNR.^67^ These maps have expanded the range of applications for DPC-STEM, making it an increasingly popular choice for materials prone to beam damage. To enable theoretical comparisons of dose efficiency across different STEM imaging methods, it is crucial to consider how noise propagates through different imaging processes. Unlike TEM, where the background level determines the noise level and allows direct evaluation of dose efficiency using conventional contrast transfer functions (CTFs), STEM imaging involves computational processing that affects noise distribution, making conventional CTFs inadequate for such comparisons. To address this, a framework using noise-normalized CTFs was developed.^68^ This method evaluates the SNR of each noniterative technique as a function of spatial frequency, providing a quantitative basis for assessing performance. Within this framework, SSB ptychography was shown to be the most dose-efficient method for sufficiently thin samples, namely two-dimensional materials, improving the detector’s contrast transfer as compared to DPC or CoM-based methods, including iDPC and iCOM. It should be noted that this framework does not include iterative reconstruction methods, such as ePIE, which fall outside the scope of the current comparison.

**Figure 5:**
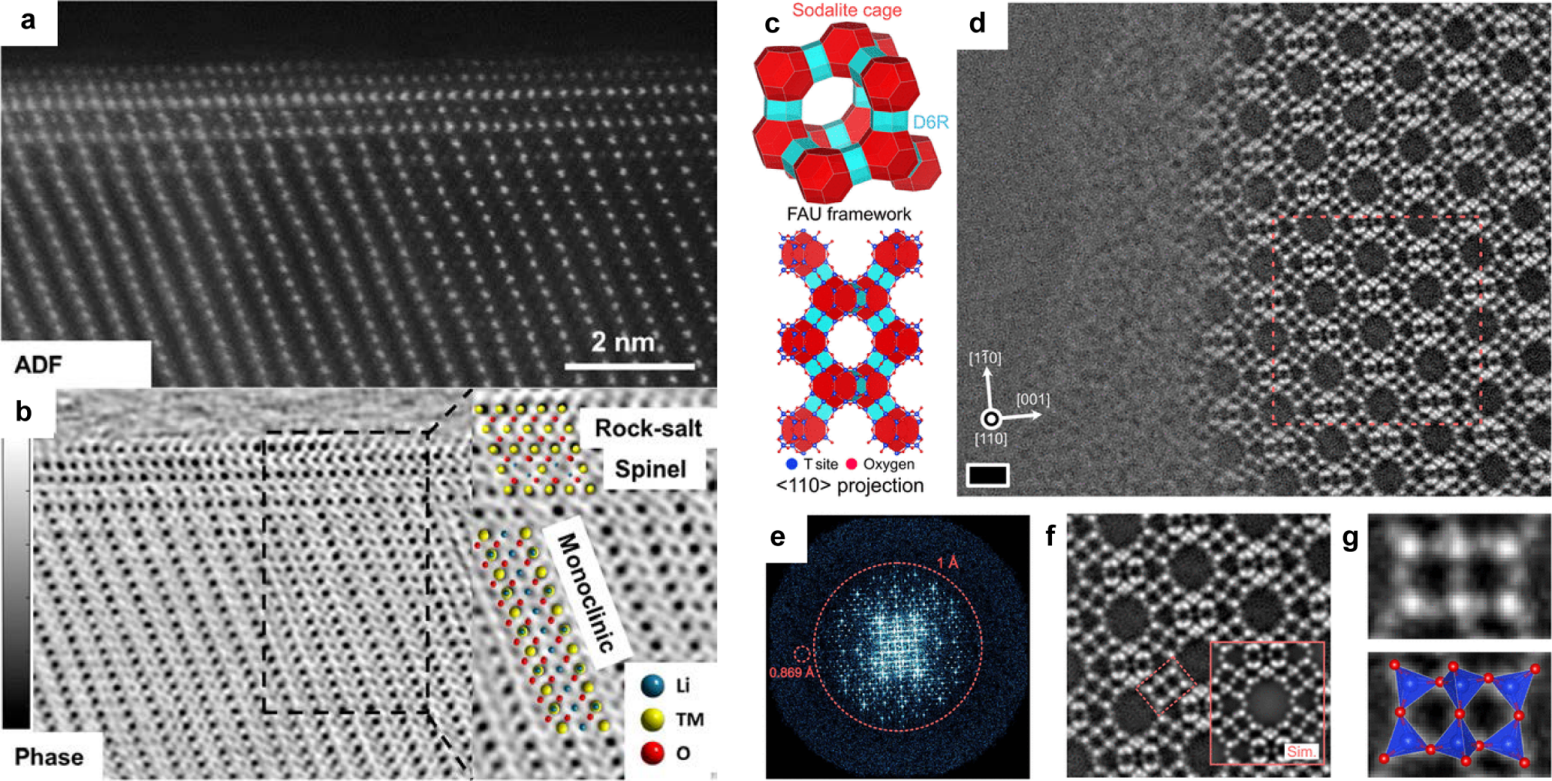
Low dose imaging applications. (a) ADF-STEM and (b) WDD ptychographic phase image reconstruction of a Li_1.2_Mn_0.6_Ni_0.2_O_2_ particle along the [010] direction, which also shows a thin layer of spinel/rock-salt along the [110] direction on the surface. The inset in (b) shows a magnified area with an atomic model superimposed indicating the positions of the transition metals (TMs), oxygen, and lithium columns. (c) Schematic of the FAU zeolite framework structure and projected atomic structure model along ⟨110⟩ zone axis. Red and blue polygons represent the building units (sodalite cages and D6Rs, respectively). (d) OBF STEM image of FAU zeolite observed at the edge of the sample. Bright spots indicate T and oxygen sites. Scale bar, 1 nm. (e) Fourier transform spectrum of (d), wherein the spots are seen up to 0.869 Å resolution in real space. (f) Repeat-unit-cell-averaged OBF image, which is obtained by cropping and averaging the multiple subimages obtained from the raw image shown in (d). The inset is a simulated OBF image calculated with the same observation condition as that in the experiment. (g) Magnified OBF image of the rectangular region indicated by the red dashed line in (f). Panels (a) and (b) reproduced from [^13^]. Copyright 2018 American Chemical Society. Panels (c)–(g) adapted from [^69^]. Available under a CC-BY 4.0 license. Copyright 2023 The American Association for the Advancement of Science.

Building on the principles of noise-normalized CTF, the OBF-STEM method was developed as a new dose-efficient imaging technique. OBF-STEM uses optimally weighted averaging of multiple STEM images acquired simultaneously with segmented or pixelated detectors to maximize SNR. A key feature of OBF-STEM is its flexibility to work with detectors of arbitrary segmentation patterns while accounting for sample thickness under the weak phase object approximation. Among dose-efficient phase imaging techniques, OBF-STEM offers the additional advantage of enabling real-time image reconstruction synchronized with electron beam scanning, which is particularly useful during microscope alignment or for rapid screening of beam-sensitive materials. OBF is a method closely related to SSB ptychography. In OBF, the sample thickness is taken into account under the weak phase object approximation, making it applicable to a wider range of conditions. As with other recent advances in phase imaging, OBF-STEM has contributed to successful imaging of challenging materials, such as imaging all atomic sites in zeolites using the real-time imaging function^69^ (Figure 5 c-g). OBF takes noise propagation into account, ensuring that the image is generated with white noise characteristics within the information limit. This approach is also employed in SSB, allowing for the suppression of artifacts while maximizing the signal.^38^ In contrast, iDPC/iCOM, which does not adopt this method, tends to emphasize noise in the low-frequency region,^69^ necessitating the use of a high-pass filter. In this context, OBF-STEM represents one of several promising approaches for high-resolution low-dose imaging.

In addition to noniterative methods like SSB or OBF-STEM, iterative ptychography has also demonstrated high dose efficiency in experimental studies.^70^ This technique is now being applied to beam-sensitive materials, further expanding its range of applications. Current implementations often utilize defocused probes to acquire 4D-STEM data, which is then processed iteratively to reconstruct phase images. While iterative ptychography requires much longer computational time than noniterative methods, its ability to extract detailed structural information by utilizing higher scattering angles, alongside potential for high dose efficiency makes it a promising approach for the future of STEM imaging.

The application of iterative ptychography has recently extended into the field of biological sample imaging, demonstrating promising results for studying delicate structures. Traditionally, biological specimens have been primarily observed using TEM. One of the most widely adopted techniques in this domain is single-particle analysis (SPA) via cryo-TEM, which has enabled structural determination of biomolecules at atomic resolution. This approach has become a cornerstone in structural biology, facilitating the visualization of proteins, viruses, and other macromolecular complexes. Phase imaging methods combined with SPA such as iDPC,^71^ ptychography^72^ and cryo-electron ptychography using iterative algorithms^73–76^ have emerged as novel methods for analyzing the structures of biological molecules. Research has shown how this latest method could have some advantages over TEM-based approaches as maintaining high resolution with an unlimited field of view or the ability to correct residual aberrations after data acquisiton.^75^ This combination of techniques has the potential to contribute to the study of biomolecules, offering high-resolution imaging with improved flexibility and addressing some limitations of traditional TEM methods.

The theoretical and experimental comparisons of dose efficiency between noniterative STEM methods, iterative ptychography, and TEM remain insufficiently explored to make conclusive statements at this stage. While recent theoretical studies suggest that TEM may achieve twice the dose efficiency of any optimal STEM experiment in ideal scenarios,^77^ these conclusions and their feasibility remain a topic of active research.^78,79^ Practical observations indicate that STEM has demonstrated unique capabilities such as the robustness of ptychographic imaging to chromatic aberrations,^80^ and the flexibility within STEM optical configurations are far from being fully investigated in this context. For example, STEM has successfully imaged samples such as zeolites,^69^ which were previously challenging for TEM, suggesting some advantages for STEM in specific cases. These findings underscore the complementary strengths of STEM and TEM in materials science. Continued research into their respective efficiencies and imaging mechanisms will provide a deeper understanding of how to best utilize these techniques. Ultimately, such advancements will help expand the boundaries of high-resolution imaging for a diverse range of materials – including many important materials subject to intense current development.

## Super-resolution

Recent advancements in STEM have enabled the surpassing of traditional resolution limits defined by the probe semiconvergence angle. The resolution of conventional imaging methods, such as HAADF imaging, is fundamentally limited to twice the semiconvergence angle (2α). Similarly, the resolution of STEM techniques based on the diffraction intensity within the bright field disk, such as iDPC/iCOM, and OBF, is also limited to 2α. However, STEM techniques utilizing diffraction patterns from high-angle scattering regions, have demonstrated the capability to achieve super-resolution well beyond this limit.^64^

Electron ptychography methods can leverage data from higher scattering angles and enable super-resolution.^80^ Super-resolution was first experimentally demonstrated by Nellist et al. three decades ago.^81^ More recently, a resolution of approximately five times the semiconvergence angle (5α) has been demonstrated using twisted bilayer MoS_2_.^82^ It is worth noting that achieving higher resolution requires the precise detection of signals from higher angle scattering regions, which consequently necessitates a significantly higher electron dose than methods utilizing the bright field region. For thicker samples, super-resolution imaging has been achieved using multislice ptychography, which accounts for dynamical scattering effects during the iterative reconstruction. This method has been successfully applied to crystalline PrScO_3_ samples up to 30 nm thick.^83^ The resulting reconstructions clearly resolved Pr–Pr atomic columns with separations as small as 59 pm, demonstrating the method’s ability to image deeply within thicker materials with atomic-scale precision. Shortly thereafter, Nguyen et al.^84^ achieved a similar resolution on a microscope without an aberration corrector (though we note this required significant computational processing time).

## Three-Dimensional Imaging and Tomography

Three-dimensional (depth sectioning) and tomographic imaging can be performed in a STEM by acquiring multiple scans while varying the defocus or the tilting of the specimen. Alternatively, an advantage of recording a 4D-STEM data set is that it allows sample interpretation beyond the simple projection approximation with a single scan, allowing some resolution along the optical axis (in contrast to the pure-projection imaging of conventional methods). In this way, we can gain image information into the third dimension. Methods enabling this three-dimensional imaging from a single 4D-STEM data set include optical sectioning and multislice electron ptychography.

Further routes to enhance three-dimensional image information can also be achieved by combining the high-contrast imaging of ptychography with STEM-tomography tilt series, in which the sample is rotated around an axis perpendicular to the beam direction, and a STEM data set collected at each sample-rotation angle.

In the early development of electron ptychography, it was noted that the 4D data set from a focused probe inherently contains some depth specific information.^80^ One can perhaps envisage this by considering a tightly focused probe passing through a thick phase object - each ray within the focused probe has passed through the specimen along a differently tilted path. The method of deconvolution of the probe from the sample with *a posteriori* chosen defocus allows a stepping through of the sample thickness. Initial development of multislice ptychography was undertaken by Maiden et al.^85^ and demonstrated with visible light. Subsequent work by Yang et al.^86^ developed and demonstrated the (electron) optical sectioning approach. In particular, it is important to note here that these approaches are not equivalent to TEM exit wave (back) propagation but enable the effects of multiple scattering to be overcome (both in theory -and demonstrated experimentally).

This approach to overcoming multiple scattering artifacts leads to a more quantitatively interpretable route to imaging the samples – we note here two particular examples of quantitative sample imaging enabled by the multislice method: Gao et al.^87^ presented the first successful implementation of this approach, while Chen et al.^83^ advanced the method further in combination with high lateral resolutions. These demonstrate a clear future direction of applications for electron ptychography.

The ability to combine ptychography with other techniques has been a clear theme in recent years. The ability to combine ptychography with tomography enables one to overcome key difficulties in imaging complex materials. This approach, first suggested by Li and Maiden,^88^ and recently demonstrated in some particularly challenging systems by Pelz et al.^89–91^ allows one to gain the full three-dimensional knowledge via a tomographic approach (taking a STEM data set at multiple sample orientations), while taking advantage of the enhanced contrast enabled by ptychographic methods. This approach promises to be particularly useful when one considers the high-dose requirements to attain ADF-STEM tomographic data sets.

## Future Perspectives

This review has shown how recent advances in phase contrast imaging methods for STEM have opened many new structural and field imaging possibilities. Observations of atomic electric fields, magnetic skyrmions and antiferromagnetic order, zeolites and biological specimens and super-resolution imaging demonstrate the technique’s versatility and potential for advancing new technologies. While challenges remain, such as observing complex samples like polycrystalline magnets and thin films, new methods such as tDPC offer promising solutions. However, there are still some unexplored areas that are difficult to observe, and, in this section, we discuss promising future developments and their applications.

The first application is to systems that could not be observed at atomic resolution due to beam damage, such as biological systems and organic materials. The use of phase imaging methods is expected to widen the application of STEM to such systems due to improved imaging efficiency under low-dose conditions. The use of ptychographic methods, including OBF, has the potential to elucidate the local structure of biological systems and organic materials from the atomic level while avoiding beam damage.

Another promising direction is the visualization of bonding electrons, a task that remains extremely difficult due to limitations in spatial resolution, instrumental stability, and residual aberrations. Nevertheless, with future advancements in instrumental stability, automated aberration correction, and detector performance, it may become feasible to detect subtle local phase shifts. This would pave the way to the direct observation of bonding electrons. Achieving such capability—particularly in localized regions such as interfaces, grain boundaries, or point defects—could provide critical insights into the atomistic origins of key physical properties, including catalytic activity, electrical conductivity, and mechanical behavior in technologically relevant systems and materials.

Another major challenge is the complete determination of three-dimensional atomic structures of local structures such as interfaces, that could be achieved by combining three-dimensional observation techniques with phase-contrast STEM methods. Phase imaging techniques are also expected to play an important role in the investigation of quantum materials, which have attracted considerable attention in recent years. In this field, structural and electromagnetic field imaging at cryogenic temperatures would be particularly valuable for elucidating the mechanisms underlying their physical properties. However, the technical hurdles for phase imaging STEM at atomic resolution under cryogenic temperatures are still high, and therefore the development of more stable liquid Ni and He holders, dedicated He stages, high-resolution magnetic field-free lenses and new aberration correction techniques are still awaited. If phase-contrast STEM observations at atomic resolution under cryogenic temperatures become widely accessible, it would open the door to the study of complex physical phenomena such as superconductivity, charge density waves, topological materials and spin related phenomena. Finally, there is the possibility of in situ observation using phase contrast STEM. Current STEM is limited in their application to in situ observation due to their limited time resolution, of just a few frames per second, which is too slow for most dynamic behaviors in materials and devices. However, the integration of recently developed high-speed scanning systems and informatics methods has led to the development of high temporal resolution STEM imaging, pushing the limit up to 25 fps TV-rates.^92^ If this high temporal resolution STEM method can be combined with phase contrast STEM, we may be able to perform TV-rate in situ phase contrast STEM imaging. Direct observation of material dynamics by phase contrast STEM at atomic dimensions is expected to show the fundamental processes of how material properties emerge. Therefore, dynamics observation by phase-contrast STEM should be an extremely powerful method for elucidating the mechanism of material properties.

In summary, continued advancements in phase imaging techniques for the STEM are expected to contribute substantially to the development of advanced materials and devices. We trust that this review can serve as a valuable resource for the community, by offering an overview of approaches to applying phase imaging methods to address scientific challenges and by providing the necessary resources to further explore these advanced characterization techniques.
